# Tailored Medication Adherence Incentives Using mHealth for Children With High-Risk Asthma (TAICAM): Protocol for a Randomized Controlled Trial

**DOI:** 10.2196/16711

**Published:** 2020-08-17

**Authors:** Brittney R Henderson, Carina M Flaherty, G Chandler Floyd, Jack You, Rui Xiao, Tyra C Bryant-Stephens, Victoria A Miller, Chris Feudtner, Chén Collin Kenyon

**Affiliations:** 1 Roberts Center for Pediatric Research Children's Hospital of Philadelphia Philadelphia, PA United States; 2 Department of Pediatrics Children’s Hospital of Philadelphia Philadelphia, PA United States

**Keywords:** asthma, pediatrics, minority, child, adolescent, metered dose inhalers, medication adherence, text messaging, financial incentives, behavior change, randomized controlled trial, clinical protocols

## Abstract

**Background:**

Poor adherence to inhaled corticosteroid medications for children with high-risk asthma is both well documented and poorly understood. It has a disproportionate prevalence and impact on children of minority demographics in urban settings. Financial incentives have been shown to be a compelling method to engage those in a high-risk asthma population, but whether adherence can be maintained by offering financial incentives and how these incentives can be used to sustain high adherence are unknown.

**Objective:**

The aim of this study is to determine the marginal effects of a financial incentive–based intervention on inhaled corticosteroid adherence, health care system use, and costs.

**Methods:**

Participants include children aged 5 to 12 years who have had either at least two hospitalizations or one hospitalization and one emergency department visit for asthma in the year prior to their enrollment (and their caregivers). Participants are given an electronic inhaler sensor in order to track their medication use over a period of 7 months. After a 1-month period of observation, participants are randomized to 1 of 3 arms for a 3-month period. Participants in arm 1 receive daily text message reminders, feedback, and gain–framed, nominal financial incentives; participants in arm 2 receive daily text message reminders and feedback only, and participants in arm 3 receive no reminders, feedback, or incentives. All participants are subsequently observed for an additional 3-month period with no reminders, feedback, or incentives to assess whether any sustained effects are apparent.

**Results:**

Study enrollment began in September 2019 with a target sample size of N=125 children. As of June 2020, 61 children have been enrolled. Data collection is estimated to be completed in June 2022, and analyses will be completed by June 2023.

**Conclusions:**

This study will provide data that will help to determine whether a financial incentive–based mobile health intervention for promoting inhaled corticosteroid use can be effective in patients with high-risk asthma over longer periods.

**Trial Registration:**

Clinicaltrial.gov NCT03907410; https://clinicaltrials.gov/ct2/show/NCT03907410

**International Registered Report Identifier (IRRID):**

DERR1-10.2196/16711

## Introduction

Poor adherence to asthma control medication is well documented yet poorly understood. Despite compelling evidence demonstrating improved disease control and fewer exacerbations with regular use of inhaled corticosteroids, adherence to prescribed inhaled corticosteroid regimens in childhood asthma has been found to be 50% of prescribed doses [1]. In minority populations living in urban settings that suffer higher rates of morbidity and mortality from asthma, adherence estimates are even lower, ranging from 11% to 37% [2-5].

Improving asthma control medication adherence, particularly in high-risk populations, remains a challenge. Previous interventions [6,7] to improve medication adherence have demonstrated effects that are modest, at best. As a result, opportunities for innovation in adherence interventions exist. Technology-enhanced reminders have demonstrated robust inhaled corticosteroid adherence improvements in countries outside of the United States [8-10]. Adult interventions consisting of financial and social incentives drawn from behavioral economics have demonstrated an effect on medication adherence and smoking cessation [11,12]. Few studies, however, have attempted to leverage mobile health technology (mHealth) and incentive design to improve medication adherence in children with high-risk asthma.

Two critical challenges have limited the efficacy of existing adherence interventions—lack of knowledge (of what it takes to engage high-risk children and their caregivers) and lack of enduring change (behavior change that lasts beyond the active intervention phase). Recent studies [13,14] have begun to identify intervention components that tend to demonstrate greater efficacy in engaging minority demographic groups and that lead to sustained changes in behavior (or habit formation). We recently conducted a pilot study [15] to investigate the effect of daily reminders and feedback supplemented with nominal financial incentives on inhaled corticosteroid adherence (US $1 per day of adherence for up to 30 days). The pilot study included families of children aged 5 to 11 years who had been hospitalized 3 times in the year prior to enrollment as a result of asthma. The proportion of participants who were approached for consent and enrolled was high (69%), and mean adherence during the intervention month (80%) was robust; however, adherence dropped to 33% after incentives, reminders, and feedback ceased [15]. These findings revealed that financial incentives were a compelling method to engage this high-risk asthma population in regular inhaled corticosteroid use; however, whether adherence can be maintained and how it can be sustained in pediatric populations is still unknown.

In this study, the intervention used in the pilot was automated by implementing the study on a behavioral research platform. This randomized controlled trial was designed to assess inhaled corticosteroid adherence trajectories in children who are at high risk for hospitalization from asthma, to identify potential mechanisms of adherence, and to assess the perceived efficacy and acceptability of the intervention among children and caregivers. The primary objective of this study is to determine the marginal effects of a financial incentive–based intervention for inhaled corticosteroid adherence on monthly adherence in children of minority demographics living in urban settings.

## Methods

### Study Design

**Figure 1:**
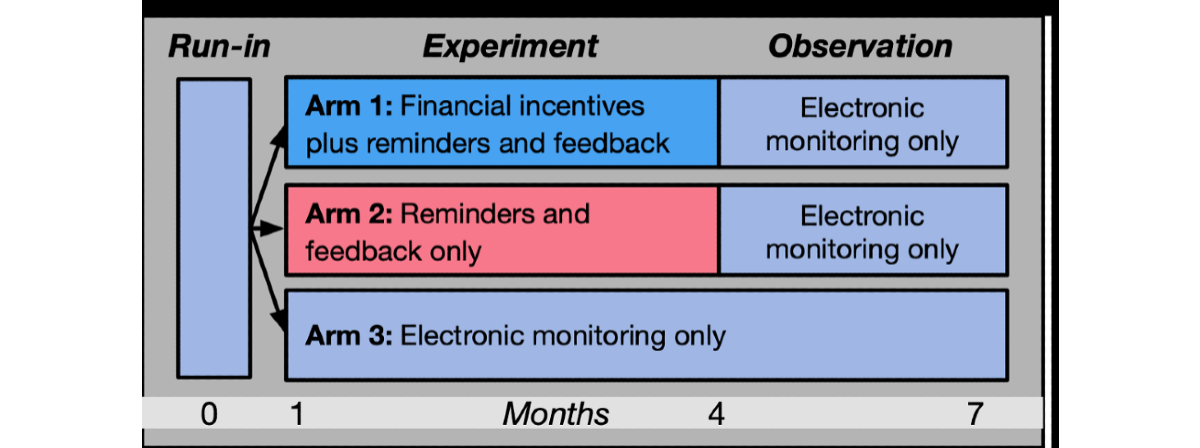
Overall study design, arm assignment, and timeline.

A 3-armed randomized controlled trial has been designed to assess the effects of different financial incentive strategies on asthma control medication adherence on a sample of children; the target population consists of children of minority demographics in urban settings with high-risk asthma. Participants are randomized to 1 of 3 experimental conditions: financial incentives, adherence reminders, and feedback (arm 1); adherence reminders and feedback without incentives (arm 2); or electronic monitoring only (arm 3). To provide an objective measure of medication use over time, upon study enrollment, research staff place electronic sensors [16] on the inhalers of participating children. These sensors have been validated and used in previous studies [17,18]. Within the study, three discrete phases span a 7-month period: a run-in phase lasting 1 month, an experiment phase lasting 3 months, and an observation phase lasting another 3 months (Figure 1). Subsequently, over a 6-month period, there is a passive follow-up phase where adherence data are not actively tracked.

### Study Setting

The study is being conducted at a large mid-Atlantic pediatric health system in the US that includes 31 primary care centers and an academic children’s hospital. Eligible participants are identified through review of daily inpatient and emergency department census reports and population health management reports in the electronic health record system.

### Inclusion and Exclusion Criteria

Children from 5 to 12 years of age (and their parent or legal guardian) who have been prescribed an inhaled corticosteroid or combination inhaled corticosteroid/long-acting beta-agonist for daily use, who have had either at least two hospitalizations or at least one hospitalization and one emergency department visit for asthma in the preceding year, whose parent or legal guardian have access to a smartphone, whose parents or legal guardians give permission (informed consent), and if appropriate, who give their assent are eligible for participation.

Participation from the study will be precluded if the mobile app is not compatible with the parent or guardian’s smartphone, their inhaler is not compatible with the electronic sensor, they have a major developmental delay or disability, they have comorbid chronic diagnoses that influences their asthma management (ie, cystic fibrosis, bronchopulmonary dysplasia, or cyanotic heart disease), their family has active Department of Human Services involvement, their family is non-English speaking, or if recruitment for participation would be against medical advice (if factors such as clinical instability or other extenuating circumstances that may influence the informed consent process are present).

Eligible children and their caregivers are recruited during an asthma-related hospital admission or emergency department visit, whenever possible, or in the month following discharge from one of these events if the study team is unable to reach the family during the hospital visit. In the latter circumstance, the study team contacts eligible participants by phone, and if the caregiver expresses interest, the study team approaches the caregiver and child for informed consent and assent at a follow-up visit or a scheduled study visit, whichever is more convenient for the caregiver. Parental or guardian permission (informed consent) and, if applicable, child assent are obtained prior to any study-related procedures being performed. Participants are enrolled through consecutive sampling until the target sample size is reached.

### Outcome Measures

The primary outcome is adherence to prescribed inhaled corticosteroid regimen during the experiment phase (months 1 to 3). Adherence is characterized as the monthly mean of daily observed-to-prescribed inhaled corticosteroid dose proportion which has an upper limit of 1; this prevents doses taken above the prescribed limit—either during flare ups or as an attempt to achieve further incentives—from influencing interval adherence measurements. Observed actuations will be quantified using Bluetooth-enabled electronic sensors (Propeller Health [16]) that attach to the inhalers. To properly track medication use from inhaler actuation, the sensors must be synced to the smartphone.

Secondary outcomes include monthly adherence to prescribed inhaled corticosteroid regimen during the observation phase (months 4 to 6), parent- and child-reported asthma control, asthma-specific health care system use and cost, and adherence trajectories. Asthma control is measured using the child Asthma Control Test [19]. Health care system use is assessed, primarily, using electronic health record data and, secondarily, from caregiver reports of visits that may have occurred outside of the institution’s health care system. Emergency department visits and hospitalizations are characterized as asthma-specific using existing, validated asthma registry definitions that include asthma visit diagnoses (ICD-9 493.XX or ICD-10 J45.XX), medication orders, and asthma pathway order set activation. Costs are estimated using average insurance payments for asthma emergency department visits and hospitalizations, in addition to the costs of the intervention components (ie, sensor, incentive, and research staff costs).

To model inhaled corticosteroid adherence over time, adherence trajectories will be constructed for the combined experiment–observation period for each participant using group-based trajectory modeling. This modeling approach accounts for changes in behavior over time and allows for identification of several distinct developmental progressions without imposing statistical assumptions of normality [20,21].

Additionally, data will be collected from semistructured interviews conducted at the end of the experiment phase in a randomly selected subset of participants. The interviews are designed to provide in-depth insight into the experiences of the child and their family with respect to intervention components, in particular, perceived influence or lack of influence of the incentives on inhaled corticosteroid adherence behavior. In addition, we will explore other factors that the children and caregivers perceive to influence daily inhaled corticosteroid adherence. Interview prompts include the perceived impact of the intervention components (financial incentives, adherence reminders, adherence feedback, study app, electronic monitoring), component design factors (duration of incentive exposure, duration and frequency of reminders and feedback, child and caregiver engagement strategies, and potential alternative incentive strategies), and nonintervention factors (medication access, perceived medication efficacy, and family and social circumstances).

### Potential Mediators

At study visits, to assess how intervention strategy impacts adherence trajectory, the following will be measured: (1) parent self-efficacy using the Parent Asthma Management Self-Efficacy scale, (2) parent and child asthma medication responsibility measured using the Asthma Responsibility Questionnaire, and (3) habit formation measured using the Habit Strength Index adapted for asthma controller use [22-24].

### Study Platform and Devices

Way to Health is a web-based intervention platform [25]. For this study, the Way to Health platform automates randomization, text message delivery, and summary of longitudinal adherence data. Text messages include reminders to administer the inhaled corticosteroid inhaler and to resync the sensor with the smartphone; reminders are sent once a week. Parents receive weekly summaries regarding medication adherence, how much money their child has earned for using their medication, and tips and encouragement for the upcoming week.

The electronic monitoring sensor records the date, time, and number of inhaler actuations. The sensor is used in conjunction with an app (compatible with iPhone and Android-based phones), which receives the medication use data from the sensor through Bluetooth. The application programming interface has been integrated with the Way to Health research platform for the purpose of this study. In order to ensure that inhaled corticosteroid use data are relayed to platform in a timely fashion, participants are instructed to ensure that their smartphone’s Bluetooth is on and that the Propeller app is opened at least once weekly, as transmission of sensor data is dependent upon these steps.

### Intervention

**Figure 2:**
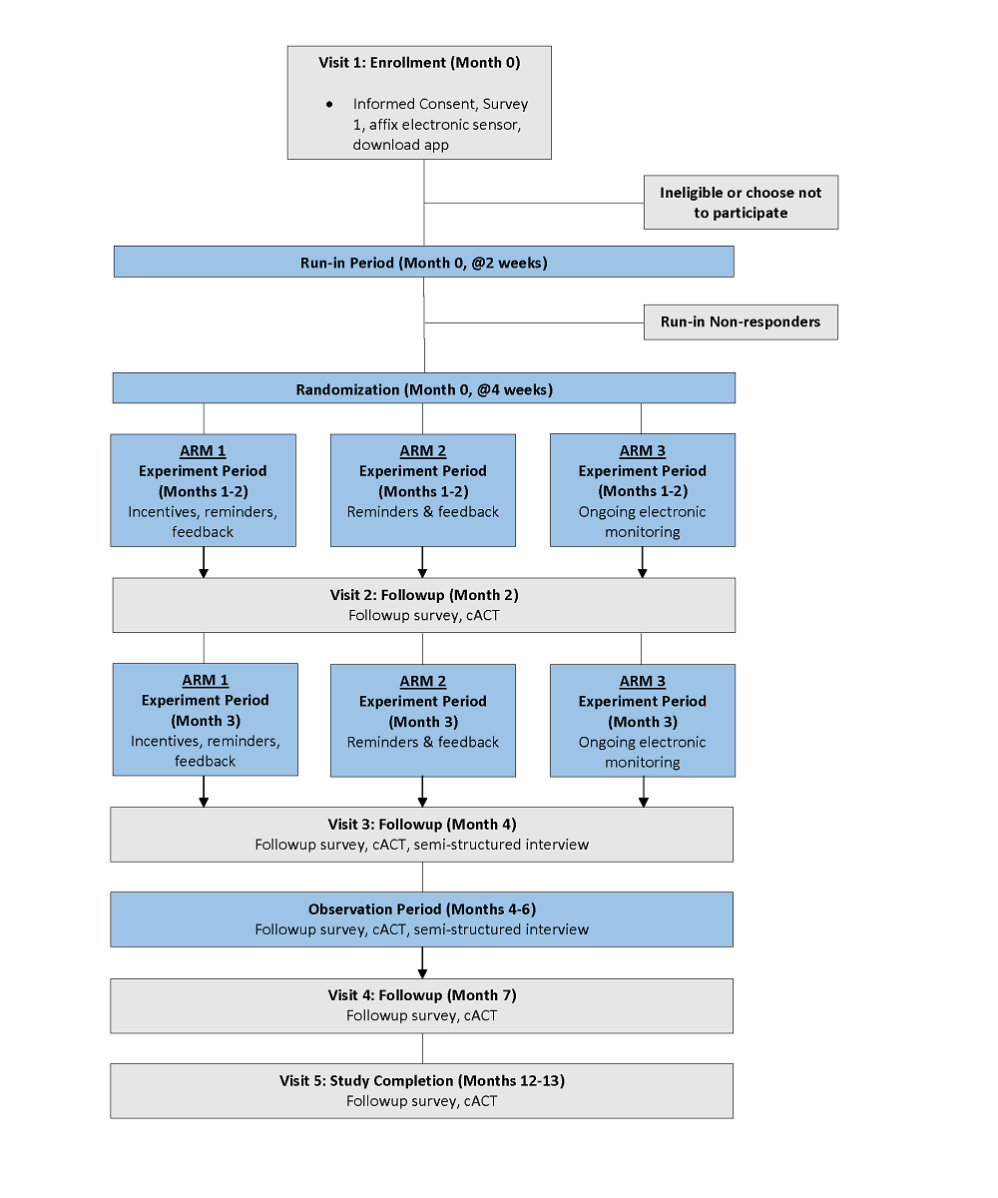
Flow diagram of the study. cACT: child Asthma Control Test.

Study visits are described in Figure 2. The first study visit consists of a 30-minute survey administered on the Way to Health platform. At the initial enrollment visit, study staff demonstrate to participants how to attach and properly sync the sensor to their inhaler and smartphone. The run-in phase begins on the day of enrollment.

During the 1-month run-in phase, all participants have access to the electronic inhaled corticosteroid use monitoring app. Inhaled corticosteroid use is tracked using the electronic sensor that remains affixed to their inhaler throughout the 7-month study period. During the run-in phase, participants are sent 4 text messages, each designed to elicit a response from the caregiver about either the study technology or the study itself. Participants for whom any inhaler use data are transmitted to the study platform within the first 2 weeks of the run-in phase AND who reply to 1 or more of the 4 text messages are randomly assigned into 1 of the 3 arms at the end of the run-in phase. Assignments are computer-generated through the Way to Health platform and are determined using a block randomization allocation sequence. The subsequent 2 weeks of the run-in phase are designed to provide baseline adherence data. Participants for whom no medication-use data are received or who do not respond to any of the text messages in the first 2 weeks are considered nonresponders and are deemed to no longer be participating in the study.

Participants are randomly allocated to arm 1 (financial incentives, adherence reminders, and feedback), arm 2 (adherence reminders and feedback without incentives) or arm 3 (electronic monitoring only) in a 2:1:2 scheme, without stratification by participant or enrollment characteristics. The financial incentives in arm 1 consist of 3 months of gain-framed, fixed-ratio incentives for each inhaled corticosteroid actuation (ie, US $0.25 per puff for children on 4 daily inhaled corticosteroid doses or $0.50 per puff for children on 2 daily doses) to a maximum of $1 per day. In arms 1 and 2, study participants receive automated daily text messages and automated weekly feedback summarizing their adherence performance through the platform.

At the end of the first month of the experiment phase, families are contacted by study staff to complete a survey that reassesses disease control, medication supply, and intentions and behaviors with regard to inhaled corticosteroid use (study visit 2). At the end of the experiment interval, families are contacted again by study staff to complete a survey (study visit 3). Participants are also randomly assigned to complete a semistructured interview for study visit 3. At this point, all reminders, feedback, and incentives cease, but daily inhaled corticosteroid use electronic monitoring continues through the observation phase (the remaining 3 months) for all arms to assess sustained effects. Surveys are complete at study visit 4 (in the final month of the study) and at the fifth and final visit at a 1-year follow-up which assesses asthma control and health care use outside of the institution’s health care system.

For participants receiving adherence incentives in the experiment phase, the accrued sum at the end of each 30-day interval (maximum $30) is added to a debit card that is provided to the child upon enrollment. In the experiment phase, children randomized to arms 2 and 3 who have any inhaled corticosteroid actuations recorded in each study month receive a $10 disbursement at the end of each month of the experiment phase (months 1 to 3). Children in all 3 study arms receive a $10 disbursement per month during the observation phase (months 4 to 6) in which any adherence data are transmitted to the platform. This compensation is not contingent upon the participant’s inhaled corticosteroid adherence. Separate study visit compensation ($20) is provided to the caregiver after enrollment and at each study visit.

After completion of the observation phase, participants enter into a follow-up period. After an additional 6 months of follow-up, participants complete a final study survey. The study team also assesses electronic health records for emergency department and hospital use for asthma over the study time period.

### Data Analysis

Baseline and demographic characteristics will be summarized as mean and standard deviation for continuous variables (ie, age) and as percentages for categorical variables (ie, gender). Study arm demographic, clinical, and past health care use variables will be compared using analysis of variance and *t* tests (or the nonparametric equivalent) for continuous variables as well as chi-square tests (or Fisher exact tests) for categorical variables.

To adjust for within-subject correlations due to repeated measures for the primary and secondary adherence outcomes, we will use generalized estimating equations that model main effects by trial arm, time (in months), and arm-by-month interactions to allow for differing inhaled corticosteroid adherence over time between arms. The dependent variable will be the mean daily observed-to-prescribed inhaled corticosteroid actuation proportion for each month. Mean daily adherence proportions are capped at 1 as previously described. All statistical tests will be 2-tailed, and a *P* value<.05 will be considered statistically significant.

We will use group-based trajectory modeling (PROC TRAJ) in SAS statistical software (SAS Institute) to construct adherence trajectories for the combined experiment and observation phases for each participant. Participants with similar daily adherence trajectories are grouped by similar longitudinal trends and the model output is probabilistic assignment of individuals to one of several adherence trajectory group models [20,21]. We will compare the models and number of groups to identify which best fit the data based on lowest Bayesian information criterion and group percentages that are sufficiently large (ie, greater than 5% of the population). A bivariate multinomial or ordered logistic regression model will be used to estimate odds ratios for the covariates and for baseline data on trajectory group membership. For the ordered logistic regression, the Brant test will be performed to check the proportional odds assumption. Variables that are not marginally predictive (*P*>.2) of any trajectory group in the bivariate analysis will be excluded from the subsequent multivariable analysis. A multivariable multinomial regression model of trajectory of all marginally significant covariates, first including baseline data, then including study arms, and finally including our 3 hypothesized mediators, will be performed [7]. Mediation analysis will be conducted with variables associated with both study arm and trajectory membership. Covariance matrices will be generated and regression estimates will be standardized to obtain an overall mediated effect of each potential mechanism variable on each of the 3 study arms. The percentage of the total effect of the study arm that is mediated by the potential mediator will be calculated using standard beta estimates [8].

For the cost offset analysis, the total medical costs will be estimated for the 12 months following study enrollment, accounting for asthma-related emergency department visits and hospitalization, as well as for expenses incurred for incentives, sensors, and the use of the Way to Health automated platform. We will estimate potential savings in medical costs, or cost offset, by calculating the difference in average total medical cost and study-related expenses for each study arm.

Semistructured interviews will be conducted with at least two trained interviewers and will be conducted in person or by phone. Audio recordings of the interviews will be transcribed by a transcription agency service and deidentified. Transcripts will then be uploaded to NVivo (version 12; QSR International LLC) for coding and analysis. At least two trained coders will use inductive theory building and the constant comparative method to identify data patterns [9,10] As themes and patterns emerge, we will search for negative cases within the data to refine our analysis [11] and create an initial set of codes that will be applied openly to a set of transcripts. Codes will be revised through an iterative process by constant comparison within a set of transcripts. A final codebook will be established and applied to all transcripts. Interrater reliability will be assessed on at least 20 percent of the transcripts to establish consistency and the coders will meet regularly throughout the process to reach a consensus on major discrepancies in coding.

### Sample Size and Power

Analysis of pilot data [15] demonstrated a 48% difference in inhaled corticosteroid adherence when comparing a time interval with financial incentives to a time period without incentives similar to the schema proposed in this study. Using a conservative estimate of detecting a 30% difference in average adherence between arm 1 (treatment arm with financial incentives corresponding to 65% adherence) and arm 3 (control arm corresponding to 35% adherence), 40 patients are required for each of these arms in the experiment phase to ensure a power of 80% at a significance level of α=.05. Estimating a 20% loss to follow-up, we aim to enroll 50 participants each in arms 1 and 3 and 25 participants in arm 2 for a total sample size of 125 participants to achieve sufficient power. This study is not powered to detect differences in relation to arm 2 (treatment arm with no financial incentives) because of insufficient existing data to estimate power, as well as practical considerations such as the number of potentially eligible patients. Rather, arm 2 will provide preliminary estimates of marginal effects of the intervention components and effect sizes on use outcomes to power a future multicenter study.

## Results

This study was approved by the Children's Hospital of Philadelphia institutional review board and is registered (Clinicaltrial.gov NCT03907410; https://clinicaltrials.gov/ct2/show/NCT03907410). Study enrollment began in September 2019. As of June 2020, 61 children have been enrolled. The intervention and follow-up phases are ongoing. Data collection is estimated to be completed in June 2022, and analyses will be completed by June 2023.

## Discussion

This study will evaluate the efficacy (using monthly inhaled corticosteroid adherence) of implementing gain-framed, nominal financial incentives, text message reminders, and weekly feedback in a cohort of children with multiple asthma exacerbations in the preceding year. While our previous work [15] demonstrated the feasibility and acceptability of financial incentives to encourage inhaled corticosteroid use among a high-risk asthma population, this study will assess the efficacy of such an approach. The study will also assess trajectories of inhaled corticosteroid adherence in the six months following the mHealth intervention to see if and how inhaled corticosteroid adherence varies between the study arms.

This study has several limitations. Participants who are adherent may still experience asthma morbidity since morbidity can be influenced by factors other than medication adherence, such as environmental exposures. This intervention will occur in the context of standard asthma care provided within our health care system; thus, children and families may be referred to a community health worker home visiting program, housing initiatives, and other programs that address social and environmental determinants of health based upon predetermined eligibility criteria. Because this study design is randomized, these exposures should not vary by study arm. Additionally, because children from 5 to 12 years of age cannot be expected to have personal smartphones to receive medication reminders, feedback, and monetary rewards, this study relies on the caregiver to relay reminders and feedback to their children. Our pilot intervention data demonstrated that most children regularly engaged with their caregiver’s smartphone, inquiring about adherence performance and incentive accrual over a 1-month period [15]. A third limitation is the challenge of implementing a technology-based intervention for high-risk families. This includes lower prevalence of home internet service, limited monthly data on cellular contracts, and the possibility that parents’ cell phone numbers may change throughout the course of the study, making it difficult to contact study participants. Additionally, some caregivers and their children will not always be in the same location and this could limit their ability to relay the reminder messages to their children. To address these limitations, we limit the frequency of adherence feedback (weekly reminders) to compensate for intermittent data uploads, and we obtain multiple telephone numbers and alternate contacts in the event that caregivers cannot be reached. Also, since having a compatible smartphone is part of the inclusion criteria, there is the potential for selection bias based on smartphone ownership. Notably, however, smartphone ownership is nearly ubiquitous, mitigating this concern [26,27]. Lastly, there may be differential attrition and data loss by study arm. Since only 1 of the 3 study arms receives financial incentives, there is a possibility that the other arms could have a higher attrition rate; however, this limitation is addressed by compensating participants in arms 2 and 3 based solely on receipt of monthly data, regardless of their adherence percentage.

## Abbreviations

ICD-9: International Classification of Diseases, Ninth Revision
ICD-10: International Statistical Classification of Diseases, Tenth Revision
mHealth: mobile health technology
